# Off-Pump Coronary Artery Bypass Graft (OCABG) Surgery Outcome: AKI Incidence, Serum Uric Acid, and Cut-Offs of Variables

**DOI:** 10.1155/2024/5945687

**Published:** 2024-07-04

**Authors:** Mohamad Reza Zare-Khormizi, Fatemeh Pourrajab

**Affiliations:** ^1^ Cardiovascular Research Center Institute of Basic and Clinical Physiology Sciences Kerman University of Medical Sciences, Kerman, Iran; ^2^ School of Medicine Shahid Sadoughi University of Medical Sciences, Yazd, Iran; ^3^ Reproductive Immunology Research Center Shahid Sadoughi University of Medical Sciences, Yazd, Iran

**Keywords:** acute kidney injury (AKI), mortality rate, off-pump coronary artery bypass grafting (OCABG), uric acid level

## Abstract

**Purpose:** One of the most important challenges of the medical community is to find out the success rate of coronary artery bypass surgery and control complications after surgery, including acute kidney injury (AKI). The present study was conducted with the aim of determining the predictive effect of serum uric acid (SUA) (UA) level in patients undergoing off-pump coronary artery bypass (OCABG) surgery.

**Methods:** The present descriptive-analytical study included 144 patients who underwent OCABG and met the inclusion criteria. SUA and related indicators, duration of hospitalization and stay in ICU, AKI and in-hospital mortality, and 6-month follow-up mortality were investigated.

**Results:** Patients were divided into high and normal groups based on SUA levels. The prevalence of postoperative AKI was 20% and was significantly associated with the preoperative UA levels (OR: 2.04; CI: 95%; 1.03–4.20). The mortality rate of patients was between 2% and 9%, which increased to 13% in patients with high SUA (*p* value ~0.224). The average duration of ICU and hospitalization in patients with high UA was longer than the other group (*p* value ~0.06 and *p* value ~0.002, respectively).

**Conclusion:** SUA levels are independently associated with a higher risk of AKI and outcome complications after off-pump CABG, and confounding factors at specific cutoffs affect the odds ratio of UA for AKI occurrence.

**Summary**

• The overall mortality rate after off-pump CABG surgery ranged from 2% to 9% and increased to 13% in patients with increased SUA levels (male > 6.5, female > 5.9).
• AKI was a common perioperative complication (frequency ~20%) and significantly increased (frequency ~33%) in patients with high SUA levels.
• SUA level was independently associated with a higher risk of in-hospital AKI in patients undergoing off-pump CABG.
• There are specific cutoffs of various variables associated with the odds ratio of UA in the incidence of AKI after OCABG surgery, including age (cutoff ~65), gender, body mass index (cutoff ~30), and glomerular filtration rate (cutoff ~60).

## 1. Introduction

Millions of patients undergo cardiac and vascular surgery every year in the world. Acute kidney injury (AKI) is a frequent and serious complication for patients undergoing both cardiac surgery and vascular surgery, occurring in 20% to 70% of cases depending on the type of surgery and the presence of risk factors. AKI in the hospital is associated with excess mortality rate and also independently associated with a higher risk of different cardiovascular events in the first year after discharge.

Duration and type of procedure of cardiopulmonary bypass (CPB) are believed as risk factors determining surgical outcome. It has been proposed that avoidance of CPB with off-pump coronary artery bypass grafting (OCABG) may reduce perioperative renal insult [1–4].

Herein, this study was conducted to investigate the mortality rate in the hospital and in the first year and the incidence rate of AKI after coronary artery bypass grafting (CABG) by the off-pump method.

In patients who are candidates for CABG coronary artery disease is commonly coexisted with atherosclerotic renal artery disease (ARAD), but with normal or near-normal baseline renal function [2, 3]. High level of serum uric acid (SUA) is found in atherosclerotic patients with cardiovascular diseases and in people with renal dysfunction and has been proposed as a risk factor in these complications [5–7].

UA levels have been associated with endothelial dysfunction, decreased nitric oxide (NO) production, and the first stage of development of coronary artery disease [6].

High SUA has been reported as an important factor in the development of the cardiorenal metabolic syndrome (CRS). The CRS is a constellation of cardiac, kidney, and metabolic disorders including insulin resistance, obesity, metabolic dyslipidemia, high blood pressure (BP), and evidence of early cardiac and kidney disease [5, 8]. It was proposed that UA levels may enhance the renin-angiotensin system (RAS), promote renin secretion, and stimulate intrakidney RAS [7].

UA levels have been shown to be associated with the cumulative incidence rates of chronic kidney disease (CKD) and that the risk of end-stage kidney disease significantly increased with higher uric acid levels, with SUA ≥ 6 mg/dL. Also, SUA above an arbitrary cut-off level (> 6.1 mg/dL) has been reported as a potential risk factor for renal insult after heart valve and aneurysm surgery [4, 7].

A relationship between serum UA, endothelial function, and small vessel remodeling in human has been reported. The addition of serum UA to the European Heart Score has been proposed to improve the identification of subjects with greater microvascular remodeling and risk of cardiac events [9].

In view of previous reports that AKI is a frequent complication following CSV, this study was designed to determine whether serum UA is an independent predictive risk factor for AKI and procedural outcomes in patients undergoing CABG surgery by off-pomp approach.

Herein, SUA levels or their relationships with other indicators of heart and kidney function can be useful markers to prognosis the patient's response to the surgery and the outcome of the treatment. We also analyzed the effect of confounding factors such as age, body mass index (BMI), glomerular filtration rate (GFR), and gender on the incidence of AKI, separately.

The findings of this research may exhibit the importance of measuring the SUA levels beside the cut-off of confounding variables before the OCABG surgery to predict possible problems and occurrence of AKI complication after the procedure.

## 2. Method of Implementing

### 2.1. Sampling and Determination of Sample Size

This research was a prospective cohort study or, on the other hand, a descriptive-analytical study, which included 144 patients who underwent OCABG and met the inclusion criteria. The Ethics Committee of Shahid Sadouqhi University of Medical Sciences (IR.SSU.Number: 18241/1/17/P) approved the study. Patients were selected nonrandomly from cardiovascular patients referred to the cardiac surgery department of Afshar Hospital in Yazd, from July 23, 2021, to November 22, 2021. Special conditions in order to measure and compare AKI complications, cardiac activity before and after OCABG surgery, the duration of the patient's ICU, and hospitalization and recovery according to existing standard criteria were evaluated.

### 2.2. Inclusion and Exclusion Criteria

Included patients were candidates for OCABG surgery, but their conditions were not urgent; they had medical records and clinical information and a history of cardiovascular disease, and they were included in the New York Heart Association functional class. Details related to the outcome of surgery were available; that is, in terms of response to surgery, they were included in the classification of the European System for Cardiac Operative Risk Evaluation (EuroSCORE), and left ventricular (LV) output was > 30%.

Excluded patients were those not suitable for OCABG: having hemodynamic instability, having distal vessels with a diameter < 1.5 mm or the vessels not being well visible, or severe calcification of the vessels. Patients who had MI in the previous 6 weeks were not included in the study. Patients with acute cardiac conditions (EF < 30%) and other acute diseases, and whose echocardiography related to LV function did not contain accurate and technically satisfactory information. Patients who could not be tracked 3–4 weeks after surgery were excluded from the study. Also, patients who had heart surgery before and had unstable angina and needed several types of surgery or additional equipment during the process were excluded from the study.

### 2.3. Measuring the Serum Level of Uric Acid

The commercial kit (uric acid, Pars Azmoun, Tehran, Iran) with an autoanalyzer (BT-3000, Italy) based on mg/dL units on the same day of sampling was used.

Hyperuricemia men: serum UA > 6.8 mg/dL (400 *μ*mol/L); hyperuricemic women: serum UA > 6.1 mg/dL (360 *μ*mol/L). Patients were divided into groups based on the serum level of UA:

▪ Group normal: UA < 6.1 mg/dL (women); UA < 6.8 mg/dL (men)
▪ Group high UA: UA > 6.1 mg/dL (women); UA > 6.8 mg/dL (men)

### 2.4. Data Collection

Demographic information and numerical data before surgery including previous medical history, risk factors of heart diseases, during surgery (such as the number of transplants, duration of surgery), and laboratory information and follow-up information were collected according to existing standards. Data such as the duration of hospitalization, the duration of ICU, the rate of AKI, and other parameters related to the activity of the heart during the hospitalization period are mentioned in the text on how to measure them (electrocardiography changes, echocardiography, and common biochemical biomarkers). Also, the GFR was calculated based on the Cockcroft–Gault equation.

### 2.5. Clinical Examination and CABG Surgery

The heart activity level of the patients was checked by myocardial contrast echocardiography (intensification of images using contrast), and the heart ejection fraction was measured, before surgery and 4 days after surgery.

All patients underwent anesthesia and surgery according to common and standard protocols.

CABG surgery procedure was done according to the off-pump method and was performed with a standard Madin sternotomy. After the opening of the pericardium, the stabilizing system (Medtronic-Utrecht Octopus System; Medtronic Inc, Minneapolis, MN) was placed in the sternal wound.

The sequence of grafting was always the left anterior descending (LAD) coronary artery first, followed by the left circumflex coronary artery and the right coronary artery. The diagonal branch and left circumflex coronary artery were revascularized using grafts from the right internal thoracic artery, while the LAD artery was revascularized by using the left internal mammary artery (LIMA). Proximal anastomoses were made on the ascending aorta, after the completion of the distal anastomoses using a continuous 6-0 polypropylene suture.

### 2.6. Evaluation of AKI and Clinical Outcomes

AKI was defined by the KDIGO consensus definition as an absolute increase in serum creatinine (Cr) level (Cr) of 0.3 mg/dL within 48 h after surgery compared to the serum Cr (1 day before the surgery), or a 1.5-fold increase in the serum Cr level after surgery compared to the baseline or need for dialysis within 7 days of enrollment.

The short-term follow-up protocol included the duration of the patient's hospitalization or being in the ICU, hospital mortality, and long-term follow-up was performed after the patients were discharged, including phone calls or 6-month visits after 6 months and the mortality rate.

### 2.7. Statistical Analysis of Data

For data with a normal distribution, the mean and standard deviation (mean ± SD) was used, and for nonnormal distribution, the median and distribution range was used. Univariate comparisons between groups were performed using the chi-square test for categorical variables and the Mann–Whitney rank-sum test for continuous variables.

Multivariate logistic regression analysis was used to apply the effect of all variables considering age, gender, BP, diabetes, BMI, smoking, and baseline Cr on the relationship between UA serum level and AKI and mortality rate, and *p* value < 0.05 was considered significant.

## 3. Results

### 3.1. Patient's Characteristics

This study included 144 patients who met inclusion criteria and were candidates for OCABG surgery. Their demographic information and clinical data at the beginning of the study and before the operation are shown in Table 1.

The prevalence of hyperuricemia in the candidates was 31% (45 out of 144 patients), and the average level of serum UA was 7.6 ± 1.2 in the high group and 4.48 ± 1.1 in the normal group. Generally, patients with increased levels of UA had older age, higher BMI, higher BP, higher serum Cr and BUN, and lower % of ejection fraction% (EF%) and GFR.

### 3.2. The Predictive Effect of UA Levels on Hospitalization Duration

The average duration of hospitalization in patients with high UA was 7 (6–11) days and in patients with normal UA was 6 (5–9) days, *p* value was ~0.002. The average length of ICU was 3.5 (2.5–4.5) days in patients with high UA and 3 (2–4) days in patients with normal UA, *p* value ~0.06 (Table 2).

### 3.3. The Predictive Effect of UA Levels on the Mortality Rates

Out of a total of 144 patients undergoing OCABG, 3 cases (2% of candidates) died in the hospital after the surgery, in which prevalence was 1% (1 case) in the normal UA group and 4.4% (2 cases) in the high UA group, *p* value ~0.181 (Table 2).

**Figure 1:**
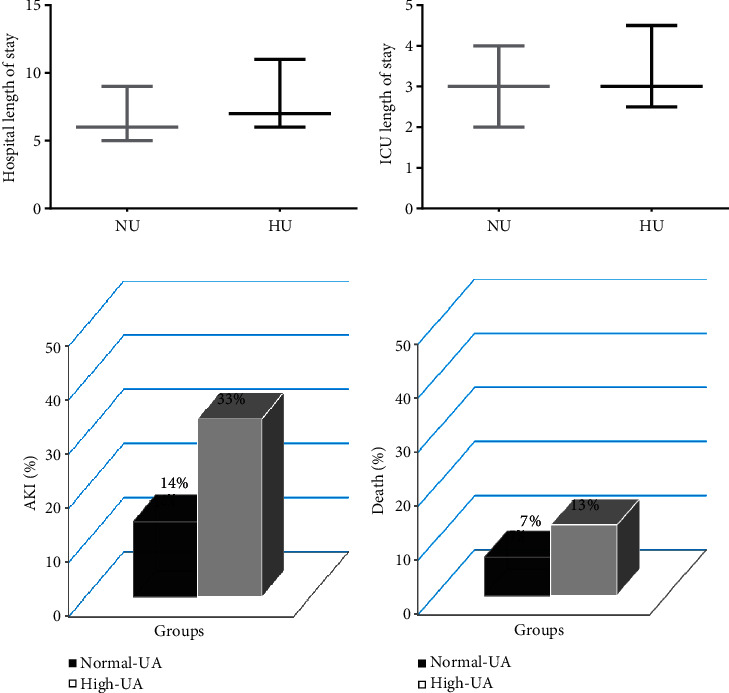
The effect of UA levels and other variables on the outcome of OCABG surgery and comparison between the patients with high UA (HU) and normal NA (NU) in the duration of (a) hospitalization and (b) ICU, (c) incidence of AKI, and (d) 6-month mortality, after OCABG surgery. (a) The average length of hospitalization in patients with high UA (HU) was 7 (6–11) and in patients with normal UA (NU) was 6 (5–9), *p* ~ 0.002. (b) The average length of ICU in patients with high UA (HU) was 3 (2.5–4.5) and in patients with normal UA (NU) was 3 (2–4) days, *p* ~ 0.06. (c) Patients with high UA levels had a higher percentage of AKI (*p* ~ 0.008) and (d) 6-month mortality rate (*p* ~ 0.224).

For investigating the mortality rate in 6 months, the follow-up protocol was performed after the discharge of the patients by phone calls or a 6-month visit. The 6-month mortality rate was 9% (13 candidates), in which prevalence was 13% (6 candidates) in the high UA group and 7% (7 candidates) in the normal UA group, *p* value ~0.224 (Table 2, Figure 1).

### 3.4. Independent and Dependent Effect of UA Levels on the Risk of AKI

**Figure 2:**
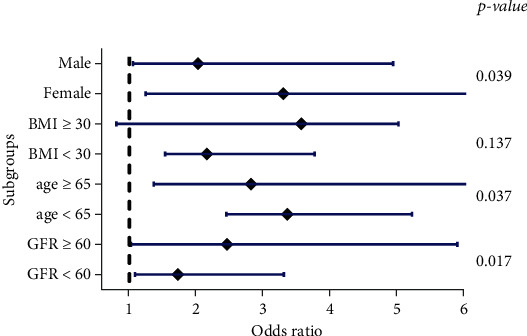
Odds ratios of UA levels are changed in specific cut-offs of different variables. Cut-offs affect odds ratios of UA in the incidence of AKI after surgery. Logistic regression analysis exhibited that the specific cut-offs of age, female gender, and GFR had significant effects on the OR of UA and AKI after surgery.

In this study, the prevalence of AKI in patients after OCABG was 20%, in which frequency was 33% in patients with high UA and 14% in the group with normal UA, *p* value ~0.008 (Table 2, Figure 2).

Without considering any other variable, a high UA level increases the risk or chance of developing AKI by 3.04 times more than a normal UA level (CI: 1.21-6.84; *p* value ~0.009).

If taking into account the effects of other variables: age, gender, BMI, hypertension, diabetes, heart failure (CHF), diuretic consumption, GFR (<60 mL/min), and Cr levels before the surgery, the risk of developing AKI by the high UA level remained significant with odds ratio (OR) ~2.04 (CI: 1.03–4.29; *p* value ~0.031) (Table 3).

The adjusted odds ratio (OR) and confidence interval (CI) of other variables affecting the development of AKI complications are shown in Table 4.

### 3.5. Specific Cut-Offs of Different Variables Has Specific Effects on the OR of UA in the Incidence of AKI

Patients were divided into different subgroups based on specific cutoffs of various variables associated with AKI, including age (cutoff ~65), gender, BMI (cutoff ~30), and GFR (cutoff ~60). Then, using the logistic regression model, the odds ratios (OR) of UA levels for the occurrence of AKI were examined (Table 5, Figure 2).

It was found that in all subgroups except BMI, preoperative serum UA level was significantly associated with AKI. The age, female gender, and GFR had significant effects on the OR of UA in the incidence of AKI after surgery. Specific cut-offs of different subgroups had significant effect on the OR of UA levels in the incidence of AKI after surgery.

Also, by dividing the patients into men and women, the results showed that UA in women has a stronger effect on the development of AKI after surgery than in men. In contrast to previous results, a stronger relationship between female gender, serum UA level, and developing AKI complications after OCABG surgery was found, in which high-UA females had a higher chance than males in developing AKI complications (Table 5, Figure 2).

In addition, by dividing the patients into two age groups, the serum level of UA and the incidence of AKI showed stronger relationships in patients over 65 years old.

## 4. Discussion and Conclusion

Studies report an overall mortality rate between 2% and 8% after heart surgery [3, 4]. A common perioperative complication for patients undergoing CSV is AKI, which is independently associated with a higher risk of different cardiovascular events in the hospital and the first year after discharge [1, 2]. Duration of the CPB procedure is considered as a potential risk factor for perioperative AKI complication. The on-pump CPB is viewed as one of the most important predictive markers for the development of postoperative AKI. Regarding renal insults, avoidance of CPB with off-pump coronary bypass has been proposed by some authors to reduce perioperative AKI complication [3, 4]. As well as, high SUA level is a common finding in patients with coronary vascular disease and has been assumed as a preoperative risk factor of AKI. According to the reports, it seems that the SUA level can be a prognostic factor for the occurrence of acute cardiac or renal events after cardiac surgery [4, 8, 10].

AKI following cardiac surgery is believed to occur due to several etiologies; major factors, including atherosclerotic renal artery disease and ischemic injury of the kidneys, resulted from inadequate perfusion. Peripheral vascular disease and coronary artery disease are commonly coexisted with unidentified atherosclerotic renal artery disease and high level of SUA, whereas renal function is initially normal or nearly normal [3, 9, 11].

In atherosclerosis and at the first stage of coronary artery disease, microvascular remodeling and endothelial dysfunction occur, which are characterized by decreased NO production and increased cytokines. SUA is one of the factors associated with endothelial dysfunction, microvascular remodeling, and an increased risk of coronary artery disease [6, 12, 13]. Increased levels of SUA have adverse effects on endothelial function and the creation of inflammatory factors, as well as, on the accumulation of platelets, all of which can be the cause of vascular remodeling and complications in atherosclerosis [5–7, 9].

SUA is associated with increased arterial pressure, inflammatory conditions, and vasoconstriction and might be a predictor of acute cardiac-renal events in those who suffer from vascular occlusion and reduced blood supply [2, 3, 14].

Mentioning UA as a predominant antioxidant molecule in the plasma and necessary and sufficient for the induction of Type 2 immune responses, increased level of SUA is an indicator of increased oxidative stress. The activity of xanthine oxidase, the most important enzyme in UA production, is a source of free radical production in cells and plasma [5–7, 9].

**Figure 3:**
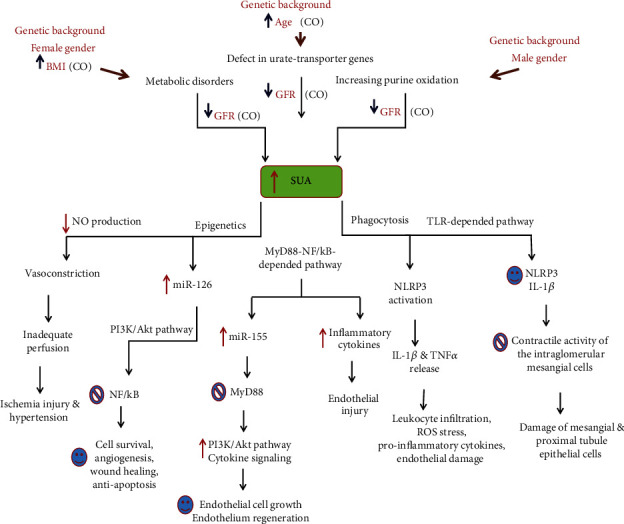
Molecular role of uric acid on the vascular remodeling in atherosclerosis and acute kidney injury. CO, cutoffs.

Different pathological mechanisms justify the relationship between increased SUA levels and cardiovascular diseases (Figure 3). UA is synthesized mainly by the liver, intestine, and vascular endothelium from oxidation of purines that are exogenous or endogenous. Damaged, dying, and dead cells release nucleic acids that lead to the production of endogenous purines [11, 14, 15].

High SUA levels can be due to both increased UA production and impaired renal UA excretion (Figure 3). Defects in the function of the genes (e.g., SLC22A and OAT) regulating urate transport and homeostasis in renal proximal tubules and endothelium can result in increased SUA levels. For example, the urate-anion exchanger urate transporter 1 (URAT1), organic anion transporter 1/3/4 (OAT1/3/4), and the glucose transporter GLUT9 regulate SUA homeostasis and urate transport. URAT1, encoded by the SLC22A12 gene, is highly expressed on the apical membrane of renal proximal tubules where reabsorbing urate. The OAT1/3/4 transporters on the renal proximal tubule membrane also control renal UA reabsorption and secretion. OAT1, OAT3, and GLUT9 are encoded by the SLC22A6, SLC22A8, and SLC2A9 genes, respectively [14, 15].

UA at high concentration is deposited as urate in endothelial cells and leukocytes that can stimulate the expression of inflammatory genes IL-1*β*, TNF-*α*, IL-6, IFN-*γ*, IL-12p40, and MCP-1 through MyD88-NF-*κ*B dependent pathway [11]. MyD88, the signal transduction adapter, links Toll-like receptor (TLR) family members and IL-1 receptor (IL-1R) to IL-1R-associated kinase (IRAK) and leads to the activation of nuclear factor kappa B (NF-*κ*B) and its nuclear translocation for the expression of proinflammatory cytokines and BCI/miR-155 [16–18].

SUA concentration has a close relationship with liver synthesis of inflammatory factors such as IL-6, TNF-*α* and CRP, and IGF-1 [2, 3, 14].

Moreover, SUA level positively affects circulating levels of leukocyte-specific miR-155, endothelial-specific miR-126, and myocardial-specific miR-499 [12, 13, 15]. Increased levels of miR-155, miR-126, and miR-499 reflect the progression of cardiovascular disease and endothelial dysfunction [12, 13]. The signature reflects the proinflammatory and prothrombotic state of atherosclerosis and indicates an urgent need for reparative procedures for the reestablishment of perfusion in coronary arteries. A significant decrease in the serum levels of miRNA signature and SUA level was observed 4 days after OCABG and vascular surgery [13, 14].

In hypoxia, the endothelium and myocardium overexpress miR-126 and miR-499, respectively, and release them into the circulation via exosomes (Exo-miR-126 and Exo-miR-499), as mediators of intercellular communication promoting angiogenesis, anti-inflammatory response, and cardiac differentiation, to maintain homeostasis [16, 19, 20]. Hypoxic myocardium releases Exo-miRNA-499 as an intercellular mediator that positively affects ventricular contractility and cardiac ejection fraction (%EF) [12, 13].

Exo-miR-126 alleviates UA-induced endothelial injury and dysfunction through upregulation of the PI3K/AKT pathway. By suppressing PIK3R2, Exo- miR-126 activates the PI3K/Akt pathway in human endothelial stem cells and accelerates angiogenesis, inhibits apoptosis, and promotes functional recovery after injury [19–21].

In suppressing inflammation, miR-126 directly targets the transcript of HMGB1 and proinflammatory chemokine MCP-1 [11, 14, 15, 19]. In endothelial injury, the alarmin HMGB1 is released and stimulates TLR to induce proinflammatory and chemotactic cytokines, IL-1*β*, IL-6, IFN-*γ*, TNF, and MCP-1 [14–16, 19].

The systemic mechanism by miR-155 is defined through the suppression of apoptosis-triggering molecules and inducing cell growth and survival. Direct targets of miR-155 are SHIP-1, SOCS-1, MyD88, HDAC, and inflammasome NLRP3. Downregulation of SHIP-1 (a phosphatase acting as an inhibitor of the PI3K pathway) and SOCS1 (a suppressor of cytokine signaling) leads to the phosphorylation of Akt and subsequent activation of the PI3K/AKT pathway [11, 15, 16].

Notably, it seems that UA initially stimulates the expression of miR-126 and the gout risk gene NRBP1 through an epigenetic mechanism involving histone deacetylation (e.g., HDAC8 overexpression) and DNA hypomethylation of transcription-factor (e.g., TFAP2A) binding sites within the promoters of regulated genes [22, 23].

Conclusively, the current study was conducted prospectively in order to investigate the duration of hospitalization, the rate of mortality, and the incidence of AKI in patients who were selected to undergo CABG surgery by the off-pump procedure. As well, the predictive effect of SUA level and the cut-off of confounding variables in AKI was investigated.

• The overall mortality rate after off-pump CABG surgery ranged from 2% to 9% and increased to 13% in patients with increased SUA levels (male > 6.5; female > 5.9).
• AKI was a common perioperative complication (frequency~20%) and significantly increased (frequency ~33%) in patients with high SUA levels.
• SUA level was independently associated with a higher risk of AKI complication in the hospital in patients who underwent off-pump CABG procedure.
• After adjusting the intervening factors using multivariate regression analysis, patients with high SUA levels compared to the rest of the patients had a multiple increase in the incidence of AKI and an increase in the duration of hospitalization.
• Using logistic regression, variables such as age, gender, BMI, BP, diabetes, CHF, use of diuretics, GFR, and creatinine levels act as intervening factors in the occurrence of AKI.
• In this study, patients were divided into different subgroups based on specific cutoffs of various variables associated with AKI, including age (cutoff ~65), gender, BMI (cutoff ~30), and GFR (cutoff ~60).
• Specific cut-offs of different variables had significant effects on the odds ratio of UA in the incidence of AKI after OCABG surgery.

Confounding factors in specific cut-offs significantly influenced the relationship between SUA and AKI (Figure 2).

## Figures and Tables

**Table 1:** Distribution of demographic and clinical variables in the study groups before the OCABG surgery.

**Variables**	**Patients % (** **n** **)**	**Serum levels of uric acid before the surgery**		
		**Normal uric acid**	**High uric acid**	**p** **values**
Numbers (%)^∗^	(100%) 144	(69%) 99	(31%) 45	—
Age (year)		59.2 ± 8.3	62.9 ± 8.1	0.015
Males (%) (*n*)	(69%) 99	(65%) 64	(78%) 35	0.115
BMI	24.9 ± 4.1	24.3 ± 3.9	26.1 ± 4.2	0.008
Smoking (%)	(21%) 31	(20%) 20	(24%) 11	0.56
Hypertension (%)	(47%) 68	(40%) 40	(62%) 28	0.01
Diabetes mellitus (%)	(26%) 37	(24%) 24	(30%) 13	0.504
Hyperlipidemia (%)	(40%) 57	(38%) 37	(44%) 20	0.44
Previous MI (weak ≥ 6)(%)	(27%) 39	(28%) 28	(24%) 11	0.63
Ejection fraction (EF%)	(40–50) 45	(45–55) 50	(40–45) 40	≤0.001
GFR^∗∗^		69.7 ± 17.6	59.3 ± 16.4	0.001
FBS		(97–141) 111	(99–153) 114	0.36
BUN	39.9 ± 15.4	37.2 ± 15.1	48.8 ± 15.7	0.005
Cr	1.08 ± 0.17	1.02 ± 0.18	1.17 ± 0.23	≤0.001
ACEI/ARB (%)	(34%) 49	(33%) 33	(36%) 16	0.79
Diuretic consumption (%)	(22%) 31	(18%) 17	(32%) 14	0.045
Patient grafts	(2–3) 3	(2–3) 3	(2–3) 3	0.01
Surgery duration (h)	(2.5–3.5) 3	(2–3.5) 3	(2.5–3.5) 3	0.32

^*^ANOVA was used for analysis.

^**^Glomerular filtration rate.

**Table 2:** Determining and comparing the frequency distribution of outcomes and complications of AKI after surgery in two groups of UA levels.

**Outcomes**	**Serum levels of uric acid before the surgery**			
	**All patients**	**Normal uric acid**	**High uric acid**	**p** **values**
Hospitalization^∗^	(6–10) 7	(5–9) 6	(6–11) 7	0.002
ICU duration (days)	(2–4) 3	(2–4) 3	(2.5–4.5) 3	0.06
AKI^∗∗^	(20%) 29	(14%) 14	(33%) 15	0.008
Hospital mortality	(2%) 3	(1%) 1	(4.4%) 2	0.181
6-month mortality	(9%) 13	(7%) 7	(13%) 6	0.224

^*^Mann-withney test was used for analysis.

^**^ANOVA was used for analysis.

**Table 3:** Determine the odds ratio (OR) between UA levels before surgery and AKI after surgery based on the logistic regression model.

**UA levels before surgery**	**Univariate analysis**		**Multivariate analysis**	
	**OR (CI:95%)**	**p** **value**	**OR (CI:95%)**	**p** **value**
Normal UA	1	—	—	1
High UA	3.04 (1.21–6.84)	0.009	2.04 (1.03–4.29)	0.031

*Note:* Using logistic regression, the effect of high uric acid serum level and normal uric acid before surgery on acute kidney complications after heart surgery alone and along with confounding variables was investigated, and the actual effect was 2.04.

**Table 4:** Odds ratios (OR) of different variables as independent risk factors in the occurrence of AKI after surgery based on multivariate logistic regression model.

**Variables**	**OR**	**CI (95%)**	**p** **value**
Age	1.20	1.07–1.35	0.002
Males	2.41	1.02–5.92	0.04
BMI	1.32	1.07–1.62	0.007
BMI > 30	1.75	0.787–4.92	0.16
Hypertension	2.05	0.863–3.44	0.12
Diabetes mellitus	1.11	0.568–2.21	0.74
GFR < 60	1.08	1.02–1.16	0.010
CHF	2.67	0.576–7.88	0.239
Cr levels	1.09	1.02–1.17	0.005
Diuretic drug	1.89	0.836–4.37	0.126
UA levels	2.05	1.26–3.63	0.014

*Note:* With logistic regression model for independent variables, age, male gender, body mass index, glomerular filtration rate (GFR), and Cr and UA levels were risk factors associated with the AKI occurrence after surgery.

**Table 5:** Specific cutoffs of different variables had specific effects on odds ratios of UA levels in the risk of AKI after surgery.

**Groups** ^∗^	**OR (CI:95%)**	**Numbers**	**p** **value** ^∗∗^
Genders			
Males	2.04 (1.07–4.95)	99	0.039
Females	3.31 (1.26–6.34)	45	
Body mass index (BMI)			
BMI ≥ 30	3.58 (0.821–5.03)	24	0.137
BMI < 30	2.17 (1.35–3.78)	120	
Age			
≥65	2.83 (1.38–6.68)	48	0.037
<65	3.37 (2.46–5.23)	96	
Glomerular filtration rate (GFR)			
≥60	2.47 (1.04–5.91)	95	0.017
<60	1.74 (1.10–3.32)	49	

^*^Based on specific risk factors related to AKI, patients were divided into different subgroups with specific cutoffs: age (cut-off ~65), gender (male or female), body mass index (cut-off ~30), and glomerular filtration rate (cut-off ~60).

^**^The chance of AKI post OCABG was determined based on serum UA level before surgery, by using the logistic regression model.

## Data Availability

Data sets used or analyzed during the current study are available from the corresponding author upon reasonable request.
